# Practical Application of DaTQUANT with Optimal Threshold for Diagnostic Accuracy of Dopamine Transporter SPECT

**DOI:** 10.3390/tomography7040081

**Published:** 2021-12-18

**Authors:** Matthew Neill, Julia M. Fisher, Christine Brand, Hong Lei, Scott J. Sherman, Ying-Hui Chou, Phillip H. Kuo

**Affiliations:** 1 Department of Medical Imaging, University of Arizona, Tucson, AZ 85724, USA; maneill484@gmail.com; 2 Statistics Consulting Laboratory, BIO5 Institute, University of Arizona, Tucson, AZ 85721, USA; julia@statlab.bio5.org; 3 General Electric Healthcare, Marlborough, MA 01752, USA; Christine.Brand@med.ge.com; 4 Department of Neurology, University of Arizona, Tucson, AZ 85724, USA; Hong.Lei@bannerhealth.com (H.L.); ssherman8@gmail.com (S.J.S.); 5 Department of Psychology, University of Arizona, Tucson, AZ 85721, USA; yinghuichou@arizona.edu; 6 Departments of Medical Imaging, Medicine, and Biomedical Engineering, University of Arizona, Tucson, AZ 85724, USA

**Keywords:** DaT-SPECT, DaTQUANT, Parkinson, ioflupane, quantification, FP-CiT

## Abstract

Evaluation of Parkinsonian Syndromes (PS) with Ioflupane iodine-123 dopamine transporter single photon emission computed tomography (DaT-SPECT), in conjunction with history and clinical examination, aids in diagnosis. FDA-approved, semi-quantitative software, DaTQUANT^TM^ (GE Healthcare, Chicago, IL, USA) is available to assist in interpretation. This study aims to evaluate the optimal variables and thresholds of DaTQUANT to yield the optimal diagnostic accuracy. It is a retrospective review with three different patient populations. DaT-SPECT images from all three study groups were evaluated using DaTQUANT^TM^ software, and both single and multi-variable logistic regression were used to model PS status. The optimal models were chosen via accuracy, sensitivity, and specificity, then evaluated on the other study groups. Among single variable models, the posterior putamen yielded the highest accuracy (84% to 95%), while balancing sensitivity and specificity. Multi-variable models did not substantially improve the accuracy. When the optimal single variable models for each group were used to evaluate the remaining two groups, comparable results were achieved. In typical utilization of DaT-SPECT for differentiation between nigrostriatal degenerative disease (NSDD) and non-NSDD, the posterior putamen was the single variable that yielded the highest accuracy across three different patient populations. The posterior putamen’s recommended thresholds for DaTQUANT are SBR ≤ 1.0, z-score of ≤−1.8 and percent deviation ≤ −0.34.

## 1. Introduction

Parkinson’s disease and related neurodegenerative disorders, such as multiple system atrophy and dementia with Lewy Bodies (DLB), demonstrate pathologic loss of nigrostriatal dopaminergic neurons. In conjunction with history and clinical examination, evaluation of nigrostriatal dopamine transporters (DaT) in Parkinsonian Syndromes (PS) can be performed with DaTSCAN^TM^ (Ioflupane iodine-123 [I123]) using single photon emission computed tomography (SPECT). DaT-SPECT imaging evaluates the dopaminergic neuronal pathway via radiotracer uptake by presynaptic dopamine transporters in the striatum [1,2,3,4,5,6]. This allows for the differentiation between nigrostriatal degenerative disease (NSDD) and other non-NSDD entities, such as essential tremor, vascular Parkinson’s, drug-induced Parkinson’s, and Alzheimer’s disease, which do not involve degeneration of dopaminergic neurons, but may present with similar clinical features.

Semi-quantitative software has been developed to assist in the interpretation of DaT-SPECT imaging using reproducible, standardized methods [7,8,9]. Semi-quantitative software can be optimized to be comparable to visual reads by experienced readers, potentially allowing inexperienced readers to increase their accuracy in everyday practice, as well as improving the confidence of experienced and inexperienced reader interpretations [10,11,12,13]. DaTQUANT^TM^ (GE Healthcare, Chicago, IL, USA) is an FDA-approved, semi-quantitative software that enables the visual evaluation and quantification of DaTSCAN^TM^ images relative to normal population databases of I-123-ioflupane uptake.

Despite the well-characterized algorithms for quantification of DaT-SPECT images, no clear guidelines for clinical practice exist for how to use the multitude of parameters for optimal diagnostic accuracy [14]. To date, there has been no published assessment of the most important quantitative parameter (or combination of parameters) to distinguish normal from abnormal, nor any ideal threshold values for quantitative parameters for DaT-SPECT. Optimizing the application of commercial semi-quantitative software for routine clinical practice would improve the value of computer-aided detection (CAD) of abnormalities to improve reader accuracy and confidence. In this study, we evaluated DaTQUANT^TM^ with I-123-ioflupane images acquired across multiple patient populations and imaging centers to develop guidelines for assisting with semi-quantitative interpretation of cases in routine practice.

## 2. Materials and Methods

### 2.1. Study Population

This is an IRB-approved, retrospective review using participants from three study groups: (1) single center, movement disorder clinic population (single center, SC); (2) participants from three multicenter, Phase 3 or 4 trials (multicenter, MC); and (3) Parkinson’s Progression Markers Initiative subjects (PPMI) Available online: http://www.ppmi-info.org (accessed on 31 December 2013). 

The single-center movement clinic population (SC) included movement disorder neurology clinic patients who underwent DaT-SPECT imaging and had at least two years of clinical follow-up. A total of 129 subjects, including 79 diagnosed with PS, were included in the analysis, with seven subjects being excluded because of an inability to quantify data due to a lack of raw tomographic data.

The multi-center population (MC) comprised a total of 309 subjects involved in three separate Phase 3 or 4 trials, including 151 with a diagnosis of PS or probable DLB (pDLB). Overall, two of the trials included 120 subjects with a clinical diagnosis of either PS or non-PS [15,16], and one trial included 189 subjects with a clinical diagnosis of either pDLB or non-DLB [17]. All subjects had one–three years of clinical follow-up.

The Parkinson’s Progression Markers Initiative subjects (PPMI) included a total of 370 subjects, which were randomly selected from both the de novo PD and healthy control populations, with 195 healthy controls and 175 diagnosed with PS after 1+ years of clinical follow-up.

### 2.2. Quantification Software

**Figure 1:**
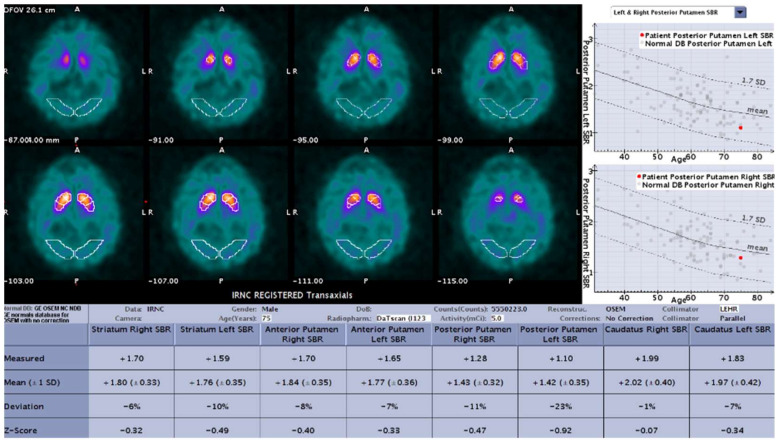
Sample result of the DaTQUANT software with the SBR (mean and deviation), z-scores, and percent deviation for multiple variables.

DaT-SPECT images from all three study groups were evaluated using DaTQUANT^TM^ software, which reconstructed all SPECT data with the same reconstruction algorithm and filter parameters as those used for the normal database and performed a volume-of-interest (VOI) determination of radiotracer binding in different regions of the striatum bilaterally. An area in the occipital cortex served as the background region. The quantified regions include striatum, caudate, putamen, anterior putamen, and posterior putamen. For each region, the striatal binding ratio (SBR: difference in mean counts between the region and background divided by the mean background counts), percent deviation from the age-matched mean of the normal database, z-score, and the age-matched mean value from the normal database is presented. Age-matching to the normal database is done on a year-by-year basis. The normal data base includes 118 healthy volunteers (no diagnosis of PS or first-degree blood relative with PS), including 73 men and 45 women, aged 31 to 84 years, who contributed to PPMI. Additional calculated values which are not background-corrected include putamen to caudate ratio (P:C), striatum asymmetry, caudate asymmetry, and putamen asymmetry (Figure 1).

### 2.3. Statistics

For each data set (MC, SC, PPMI) and DaTQUANT^TM^ summary measure (z-score, SBR, percent deviation), the ability of different combinations of VOIs to predict NSDD is evaluated where the summary measures for each VOI are the predictor variables. For each dataset, the VOIs, described previously in quantification software section above, were used with the exception of striatum asymmetry, which was not present in the MC group. For each participant and each VOI, the “more affected side” was defined as the one with the lower z-score. Only the summary measure for the more affected side is used in the modeling. Note that this means that the more affected side was not forced to be consistent across VOIs.

The binary diagnosis was modeled with logistic regression. Additionally, since prediction from logistic regression requires setting a threshold at or above which a participant is predicted to have a positive outcome (i.e., have PS/DLB), 21 equally spaced thresholds in probability space were considered, ranging from 0 to 1 by increments of 0.05. For each model and threshold combination, prediction (accuracy, sensitivity, specificity) via leave-one-out cross validation were evaluated. Two-sided bootstrap case cross validation 95% confidence intervals (1000 bootstrap data sets) are provided for the leave-one-out cross validation accuracies, a slight modification to the one-sided confidence intervals in Jiang et al., 2008 [18].

Using the results from the leave-one-out cross validation, optimal models for each combination of predictors, summary measure, and threshold were identified. These models were refit to the full data sets to get the model equations, and then evaluated on the other data sets. For this study, 95% Wilson confidence intervals for cross-group accuracies are provided [19].

## 3. Results

### 3.1. Demographics

**Figure 2:**
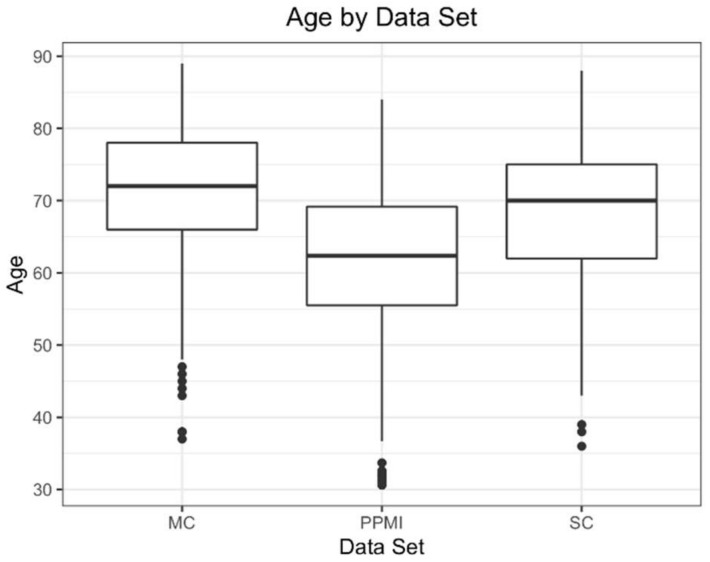
Box and whisker plots for all three data sets.

The mean age of the participants overall was 66 years old, with 40% female participants. The mean age for each individual group ranged from 62 (PPMI) to 71 (MC) years old, with 33% (PPMI) to 48% (SC) female participants (Table 1). A majority of participants were in their sixth to eighth decades of life, with the youngest participant being 36 years old and the oldest being 88 years old (Figure 2).

### 3.2. Single Variate Models

In single variable analysis for all groups, the posterior putamen of the more affected side demonstrated the highest accuracy, while still maintaining a sensitivity and specificity above 0.80 (Table 2). Overall, there were no substantial differences in accuracy, sensitivity, or specificity between the SBR, z-score, or percent deviation variables within each group (differences ranged from 0.01 to 0.03 for the different summary measures). The PPMI models overall had the highest accuracy (0.95), followed by SC (0.91), then MC (0.85). The optimum thresholds for the discussed SBR, z-score, and percent deviation models are shown in Table 2.

For all three variables in the SC group (SBR, z-score, and percent deviation), the posterior putamen demonstrated the highest accuracy, 0.90–0.91, with a sensitivity and specificity of 0.91–0.92 and 0.88, respectively. Three other regions (putamen, anterior putamen, and striatum) had identical accuracy with small (up to 0.06) differences in sensitivity and specificity, but gain in one was coupled with similar loss in the other. Additionally, multiple thresholds for the posterior putamen region demonstrate identical results for accuracy. For example, the posterior putamen SBR variable threshold value of ≤0.95 yields the same accuracy as using a threshold value of ≤1.00. For the MC group, the posterior putamen alone demonstrates the highest accuracy for all three variables (0.84–0.85), with balanced sensitivity and specificity (0.80–0.82 and 0.88–0.89, respectively). For the z-score variable, use of a threshold value of −1.52 instead of −1.73 yields identical accuracy, with 0.03 increase in sensitivity while reducing specificity by 0.03.

For the PPMI group, the posterior putamen alone demonstrates the highest accuracy for all three variables (0.94–0.95), with balanced sensitivity and specificity (0.91–0.92 and 0.95–0.97, respectively). No additional threshold models were identical for this group.

### 3.3. Multi-Variate Models

Multi-variable models did not substantially improve the accuracy, sensitivity, or specificity of the three groups, with most of the models resulting in 0.01–0.04 differences in accuracy, sensitivity, or specificity (Table 3). Several models showed increased specificity (SC group: SBR and percent deviation, MC group: SBR), but only improved the specificity by 0.03–0.04, and sacrificed up to 0.03 in sensitivity.

### 3.4. Cross Group Results

When the best models for each group were tested on the remaining two groups, the models yielded very similar results to the within-group analysis for each (Table 4). While some models showed minor differences (0.01–0.05) in certain results, the differences in improvement in one result were coupled with similar sacrifices in another result, such as sensitivity for specificity. While variables other than posterior putamen had identical accuracy results for the SC group, when these models were tested on the other groups, significant drops in accuracy were seen. For example, the SC striatum SBR accuracy when tested on the PPMI group is 0.85 versus 0.95 for the PPMI posterior putamen SBR model.

## 4. Discussion

In single-variable evaluation of optimal variables and thresholds in DaTQUANT^TM^, the posterior putamen consistently yields the highest accuracy in diagnosis. The critical importance of the posterior putamen correlates with the expected natural history of the disease process [20]. Furthermore, the single-variable posterior putamen models performed similarly when tested across all study groups, providing evidence that the models perform consistently across different patient populations and different clinical settings. While the putamen, anterior putamen, and striatum models yielded identical accuracy for the SC group, they experienced slight to moderate losses of accuracy across other groups, suggesting that these models are not quite as robust and therefore not as useful in a clinical environment.

In determining the recommended single variable threshold values for each summary measure, the SC and PPMI results were more heavily weighted, given that their patient populations are more reflective of the standard patient population undergoing DaT-SPECT in the United States of America, namely evaluation for tremor rather than dementia (which was prevalent in the MC group). With this in mind, the recommended threshold values for DaTQUANT^TM^ posterior putamen are: SBR of ≤1.0, z-score of ≤−1.8, and percent deviation of ≤−0.34.

When comparing each group’s highest accuracy single-variable model, the results differed substantially, with the highest accuracy seen in the PPMI group (0.95), and the lowest accuracy seen in the MC group (0.85). This can likely be attributed to differences in patient population. The PPMI is an observational study that has recruited normal age-matched controls (with neither movement disorder nor other indication of a PS), and a study population of PD subjects with imaging performed under clinical research protocol standards, which included exclusion of movement disorder patients without evidence of dopaminergic deficits (SWEDD). Meanwhile the SC and MC groups reflect a patient population similar to what is seen in everyday practice; thus their results may more closely reflect “real-world” accuracy of DaT-SPECT imaging.

When comparing the three summary measures SBR, z-score, and percent deviation, each measure performed similarly for all models and across all study groups. These results would allow for the interpreting radiologist to use either variable preferentially without significant differences in accuracy, sensitivity, or specificity. Additionally, while the optimal threshold values for each summary measure have small differences (for instance, optimal posterior putamen z-score thresholds from each of the three groups are ≤−1.9 vs. −1.73 vs. −1.68), each model’s optimal threshold performed similarly across all three study groups (Table 4). This consistency across different study groups suggests that, while the exact threshold values of a specific model may not be the optimal value for other patient cohorts, it performs in a similar enough fashion to be of clinical use.

A multi-variable approach did not substantially improve results. Many of the best multi-variable models in the three participant groups incorporated some form of asymmetry variable (striatum, caudate, or putamen asymmetry) without substantial improvement in accuracy, sensitivity, or specificity. Asymmetry is a prominent feature of neurodegenerative disorders, particularly Parkinson’s disease [21,22], but these findings suggest that asymmetry is not a reliable predictor overall, and focus should instead be placed onto the more affected side rather than primarily comparing sides.

Limitations and caveats to the use of the threshold provided by this study include the inclusion of movement disorder and dementia patients, as well as the age range of the patients included. The populations of the SC/PPMI and MC groups differed in one distinct, important way: the SC/PPMI groups consisted of movement disorder patients only, while the MC group consisted of movement disorder and dementia patients. Known DaT-SPECT imaging differences between patients presenting with different symptoms (tremor versus dementia) may lead to different predictive models or quantification thresholds [23,24,25]. Additionally, the age range of patients within this study should be noted, with a majority of patients in their sixth to eighth decades of life. It is known that normal loss of nigrostriatal dopaminergic neurons occurs with age [26]. Patients at either extreme of age, young and old, should be evaluated with caution, as the quantitative software results and associated predictive models may not provide similar accuracy. Furthermore, all three groups used the same reconstruction algorithm with associated parameters and normal database for determining z-score and percent deviation. Any change in the underlying reconstruction or normal database used to calculate these measures will impact the accuracy of the provided models/thresholds.

Further research areas include difficult to read cases and dementia versus movement disorder cases. The difference between movement disorder and dementia patients would benefit from further study to maximize benefit to practices that use DaT-SPECT in the evaluation of dementia. Additionally, there may be a subset of cases that are particularly hard to read and could benefit from multi-variable modeling. Further investigation into difficult or borderline cases may require a more sophisticated approach. Future research may include the use of deep learning algorithms, such as convolutional neural networks (CNN) to further optimize sensitivity and specificity. Such a method would not be restricted to pre-specified regions of interest and their derived parameters. Furthermore, the integration of clinical information may be necessary to push beyond the limitations of imaging alone.

**Figure 3:**
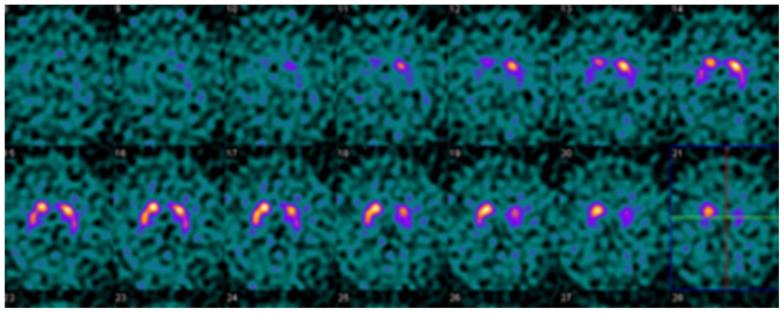
Example DaTQUANT results in a non-PS patient demonstrating asymmetric loss of uptake within the left posterior putamen. Despite the asymmetry, none of the summary measures, SBR (1.39), z-score (−0.31), or percent deviations (−0.07) meet the threshold for abnormal.

**Figure 4:**
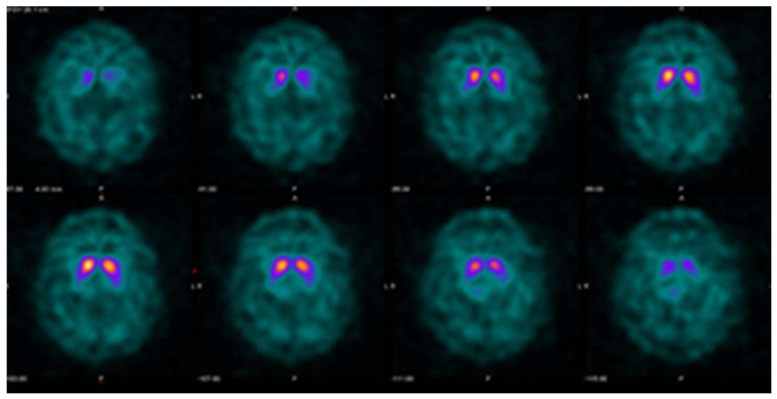
Example DaTQUANT results in a non-PS patient demonstrating apparent loss of the normal “comma shape”, particularly on the right. However, none of the summary measures, SBR (1.23 to 1.33), z-score (−0.51 to −0.88), or percent deviations (−0.12 to −0.19) meet thresholds for abnormal. The caudate nuclei are noted to have relatively increased uptake (SBR 3.10–3.45, z-score 2.42–3.48) causing an apparent decrease in putaminal activity where there is none (“hot caudate” sign).

**Figure 5:**
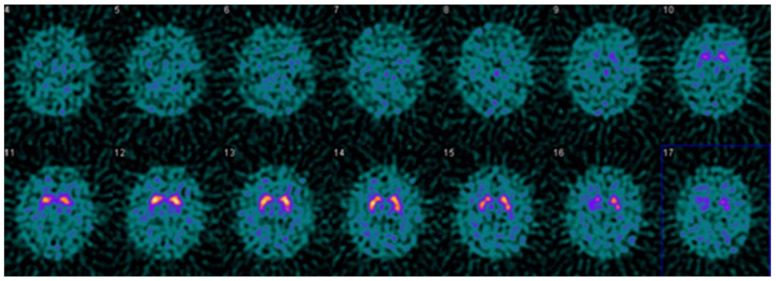
Example DaTQUANT results in a non-PS patient demonstrating visually normal appearing striatum bilaterally. However, the right posterior putamen z-score is −2.15, meeting the threshold for a diagnosis of PS. This is an example of false-positive quantification, reinforcing the idea that quantification is a useful tool for interpretation, but should not replace visual evaluation. Interestingly, the SBR (1.27) does not meet the threshold for PS, and the percent deviation (−0.35) is right at the threshold value for the optimal model (≤−0.36).

It is helpful to keep in mind that quantitative evaluation should not be used in isolation. Evaluating study quality and identifying potential artifacts may prevent mistakes in interpretation [27]. Example cases are provided in Figure 3, Figure 4 and Figure 5, illustrating the usefulness and limitations of quantification using the single variable models and thresholds in this study.

## 5. Conclusions

In typical utilization of DaT-SPECT for differentiation between NSDD and other non-NSDD, the posterior putamen was the single variable that yielded the highest accuracy across three different patient populations. For SPECT data acquired and reconstructed per the DaTQUANT^TM^ procedure specified for the IRNC database, the posterior putamen’s recommended threshold values for DaTQUANT^TM^ are SBR ≤ 1.0, z-score of ≤−1.8 and percent deviation ≤ −0.34. Multi-variable models did not substantially improve accuracy.

## Figures and Tables

**Table 1:** Demographic data of the three study groups.

Patient Characteristic	SC	MC	PPMI	Total
Mean Age (years)	67 ± 11	71 ± 10	62 ± 10	66 ± 11
Gender				
Male	67 (52%)	169 (56%)	249 (67%)	485 (60%)
Female	62 (48%)	134 (44%)	121 (33%)	317 (40%)

**Table 2:** Single Variable Results.

Study Group	Summary Measure	Variable; Threshold	Accuracy	Sensitivity	Specificity
SC	SBR	Post. Putamen *; ≤0.94 ^†^	0.91 [0.85, 0.95]	0.92	0.88
	z-score	Post. Putamen; ≤−1.9	0.90 [0.83, 0.96]	0.91	0.88
	Percent Deviation	Post. Putamen ^‡^; ≤−0.36 ^§^	0.90 [0.84, 0.95]	0.91	0.88
MC	SBR	Post. Putamen; ≤0.90	0.85 [0.80, 0.90]	0.82	0.88
	z-score	Post. Putamen; ≤−1.73 ^‖^	0.84 [0.80, 0.89]	0.80	0.89
	Percent Deviation	Post. Putamen; ≤−0.39	0.85 [0.81, 0.89]	0.82	0.89
PPMI	SBR	Post. Putamen; ≤1.01	0.95 [0.92, 0.97]	0.91	0.97
	z-score	Post. Putamen; ≤−1.68	0.94 [0.91, 0.96]	0.91	0.95
	Percent Deviation	Post. Putamen; ≤−0.32	0.94 [0.91, 0.97]	0.92	0.95

The accuracy, sensitivity, and specificity for each group’s best single variable model for each summary measure (SBR, z-score, and percent deviation) with the corresponding threshold value. If the posterior putamen value is less than or equal to the indicated threshold, the patient is predicted to have Parkinson’s disease. *: Striatum and anterior putamen models result in accuracies of 0.91 with sensitivities of 0.89–0.91 and specificities of 0.90–0.94. ^†^: Threshold of ≤1.05 resulted in accuracy of 0.91 with sensitivity of 0.94 and specificity of 0.86. Threshold of ≤1.00 resulted in identical results to ≤0.94. ^‡^: Anterior putamen and striatum models demonstrate identical results. ^§^: Thresholds of ≤−0.36 to −0.42 demonstrate identical results. ^‖^: Threshold of ≤−1.52 results in identical accuracy (0.84) with sensitivity of 0.83 and specificity of 0.86.

**Table 3:** Multi-variable results: The accuracy, sensitivity, and specificity for each group’s best multiple variable models, including three or fewer variables.

Study Group	Summary Measure	Variables	Accuracy	Sensitivity	Specificity
SC	SBR	Putamen, Post Putamen, Caudate Asymmetry	0.92 [0.85, 0.98]	0.92	0.92
	z-score	Putamen, Post Putamen, Putamen Asymmetry	0.91 [0.84, 0.96]	0.92	0.88
	Percent Deviation	Striatum, Post Putamen, Striatum Asymmetry	0.92 [0.86, 0.98]	0.92	0.92
MC	SBR	Striatum, Post Putamen	0.85 [0.81, 0.90]	0.79	0.91
	z-score	Striatum, Caudate	0.85 [0.81, 0.89]	0.80	0.90
	Percent Deviation	Post Putamen, Putamen Asymmetry	0.85 [0.81, 0.89]	0.82	0.89
PPMI	SBR	Striatum, Caudate, Putamen Asymmetry	0.95 [0.93, 0.98]	0.92	0.98
	z-score	Striatum, Caudate, Striatum Asymmetry	0.95 [0.92, 0.97]	0.92	0.97
	Percent Deviation	Post Putamen, P:C	0.95 [0.92, 0.97]	0.91	0.98

**Table 4:** Cross-group single variable results.

Study Group	Variable; Summary Measure Threshold	SC Test			MC Test			PPMI Test		
		Acc	Sens	Spec	Acc	Sens	Spec	Acc	Sens	Spec
**SC**	Post. Putamen *; SBR ≤ 0.94 ^†^	**0.91 [0.85, 0.95]**	**0.92**	**0.88**	0.83 [0.79, 0.87]	0.83	0.84	0.94 [0.91, 0.96]	0.89	0.97
	Post. Putamen; z-score ≤ −1.9	**0.90 [0.83, 0.96]**	**0.91**	**0.88**	0.85 [0.80, 0.88]	0.78	0.91	0.92 [0.89, 0.95]	0.89	0.95
	Post. Putamen ^‡^; % Deviation ≤ −0.36 ^§^	**0.90 [0.84, 0.95]**	**0.91**	**0.88**	0.86 [0.82, 0.90]	0.83	0.89	0.94 [0.91, 0.96]	0.91	0.95
**MC**	Post. Putamen; SBR ≤ 0.90	0.90 [0.84, 0.94]	0.90	0.90	**0.85 [0.80, 0.90]**	**0.82**	**0.88**	0.93 [0.90, 0.95]	0.87	0.97
	Post. Putamen; z-score ≤ −1.73 ^‖^	0.89 [0.83, 0.93]	0.91	0.86	**0.84 [0.80, 0.89]**	**0.80**	**0.89**	0.93 [0.90, 0.95]	0.91	0.95
	Post. Putamen; % deviation ≤ −0.39	0.90 [0.84, 0.94]	0.91	0.88	**0.85 [0.81, 0.89]**	**0.82**	**0.89**	0.94 [0.91, 0.96]	0.91	0.96
**PPMI**	Post. Putamen; SBR ≤ 1.01	0.91 [0.85, 0.95]	0.94	0.88	0.82 [0.77, 0.86]	0.83	0.80	**0.95 [0.92, 0.97]**	**0.91**	**0.97**
	Post. Putamen; z-score ≤ −1.68	0.89 [0.83, 0.93]	0.91	0.86	0.84 [0.80, 0.88]	0.80	0.88	**0.94 [0.91, 0.96]**	**0.91**	**0.95**
	Post. Putamen; % deviation ≤ −0.32	0.89 [0.83, 0.93]	0.92	0.84	0.83 [0.79, 0.87]	0.83	0.84	**0.94 [0.91, 0.97]**	**0.92**	**0.95**

The accuracy, sensitivity, and specificity of each group’s best single variable model when tested on the other two groups (bolded numbers are same leave-one-out cross validation group results per Table 2). 95% confidence intervals for the cross-group accuracies are Wilson confidence intervals. *: The striatum, anterior putamen, and putamen models that had similar results on the SC group resulted in drops in accuracy (0.85–0.91 for PPMI) and sensitivity (0.71–0.87 PPMI). ^†^: Threshold values of SBR ≤ 1.00 and ≤1.05 demonstrate similar results, although had lower specificity on the MC group (0.78—0.81). ^‡^: The anterior putamen model that had similar results on the SC group resulted in drops in accuracy (0.88–0.89 PPMI) and sensitivity (0.79–0.82 PPMI). ^§^: Thresholds of % deviation ≤ −0.39 and ≤−0.42 had similar results to the threshold provided in the table (within 0.01–0.02) except for ≤−0.42 sensitivity for MC (0.79 vs. 0.83). ^‖^: Threshold value of z-score ≤ −1.52 demonstrated similar results (within 0.02) except for specificity on the SC group (0.82 vs. 0.86).

## Data Availability

Data cannot be made publicly available for multiple reasons. Part of the data belongs to General Electric Healthcare and is not for public use. Part of the data from the University of Arizona belongs to the health network and the authors do not have permission to release for public use. The PPMI data should be requested from the Michael J. Fox Foundation.
